# Mechanotransduction-Driven Modulation of *L*-Type Calcium Channels: Roles of Nitric Oxide, S-Nitrosylation, and cGMP in Rat Ventricular Cardiomyocytes

**DOI:** 10.3390/ijms26157539

**Published:** 2025-08-04

**Authors:** Olga V. Kamkina, Anastasia S. Rodina, Andre Kamkin, Andrei S. Bilichenko, Viktor E. Kazansky, Alexandra D. Zolotareva, Valentin I. Zolotarev, Stanislav A. Shileiko, Vadim M. Mitrokhin, Mitko I. Mladenov

**Affiliations:** Institute of Physiology, Pirogov Russian National Research Medical University, 117997 Moscow, Russia; kamkina_ov@rsmu.ru (O.V.K.); rodina_as@rsmu.ru (A.S.R.); andrey.kamkin@rsmu.ru (A.K.); bilichenko_as@rsmu.ru (A.S.B.); kazanskii_ve@rsmu.ru (V.E.K.); zolotareva_ad@rsmu.ru (A.D.Z.); zolotarev_vi@rsmu.ru (V.I.Z.); shileiko_sa@rsmu.ru (S.A.S.); mitrokhin_vm@rsmu.ru (V.M.M.)

**Keywords:** *L*-type Ca^2+^ channels, cardiomyocytes, mechanosensitivity, nitric oxide, S-nitrosylation, NO-cGMP

## Abstract

*L*-type Ca^2+^ channels, particularly Ca_V_1.2, play a crucial role in cardiac excitation-contraction coupling and are known to exhibit mechanosensitivity. However, the mechanisms regulating their response to mechanical stress remain poorly understood. To investigate the mechanosensitivity and nitric oxide (NO)-dependent regulation of *L*-type Ca^2+^ channels in rat ventricular cardiomyocytes, we used RNA sequencing to assess isoform expression and whole-cell patch-clamp recordings to measure *L*-type Ca^2+^ current (*I*_Ca,L_) under controlled mechanical and pharmacological conditions. RNA sequencing revealed predominant expression of Ca_V_1.2 (TPM: 0.1170 ± 0.0075) compared to Ca_V_1.3 (0.0021 ± 0.0002) and Ca_V_1.1 (0.0002 ± 0.0002). Local axial stretch (6–10 μm) consistently reduced *I*_Ca,L_ in proportion to stretch magnitude. The NO donor SNAP (200 μM) had variable effects on basal *I*_Ca,L_ in unstretched cells (stimulatory, inhibitory, or biphasic) but consistently restored stretch-reduced *I*_Ca,L_ to control levels. Ascorbic acid (10 μM), which reduces S-nitrosylation, increased basal *I*_Ca,L_ and partially restored the reduction caused by stretch, implicating S-nitrosylation in channel regulation. The sGC inhibitor ODQ (5 μM) decreased *I*_Ca,L_ in both stretched and unstretched cells, indicating involvement of the NO–cGMP pathway. Mechanical stress modulates *L*-type Ca^2+^ channels through a complex interplay between S-nitrosylation and NO–cGMP signaling, with S-nitrosylation playing a predominant role in stretch-induced effects. This mechanism may represent a key component of cardiac mechanotransduction and could be relevant for therapeutic targeting in cardiac pathologies involving mechanically induced dysfunction.

## 1. Introduction

*L*-type Ca^2+^ channels are essential for cardiac function and mediate the predominant pathway for Ca^2+^ entry during the action potential of cardiomyocytes. Among these channels, Ca_V_1.2 plays a particularly important role by allowing Ca^2+^ entry during the initial Ca^2+^ influx phase that initiates Ca^2+^ release from the sarcoplasmic/endoplasmic reticulum, which in turn initiates contraction of the cardiomyocyte [1]. Ca_V_1.2 channels are predominantly localized within the T-tubule system associated with the sarcomeric Z-lines, where they are in close proximity to ryanodine receptors on the sarcoplasmic reticulum [1]. Such an arrangement allows high efficiency in excitation-contraction coupling because of rapid calcium-induced calcium release [1]. This Ca^2+^-induced Ca^2+^ release is crucial for the coherence of the excitation-contraction coupling and heart function [1].

Recent studies have demonstrated that *L*-type Ca^2+^ channels possess significant mechanosensitivity, and their gating properties are modulated by mechanical stress. Studies have shown that Ca_V_1.2 channels exhibit both single-channel and whole-cell current mechanical sensitivity [2]. Similarly, Ca_V_1.3 channels have been found to be mechanosensitive [3], but their expression in ventricular myocytes is markedly lower than in Ca_V_1.2. This mechanosensitivity places these channels in a separate category from mechano-gated channels (MGCs), and they are called mechano-sensitive channels (MSCs). A mechano-sensitive channel (MSC) is often considered any ion channel that is mechanically gated by forces such as membrane tension, cytoskeletal deformation, or extracellular matrix interactions. It should to be noted that another subclass of MSCs—mechanically gated channels (MGCs)—are directly opened by membrane stretch or pressure. This difference enables us to distinguish channels regulated indirectly by mechanical forces from channels that are inherently gated by such forces.

The regulation of *L*-type Ca^2+^ channels by nitric oxide (NO) is a complex and controversial field. It has previously been demonstrated that NO regulates the channel through two separate pathways that include the classic NO-sGC-cGMP pathway and direct S-nitrosylation of the channel proteins. The action of NO on basal Ca^2+^ currents (*I*_Ca,L_) has been described as activation, inhibition, absence of effect, or concentration dependence of the effect [4]. This wide variety of responses indicates complex regulatory mechanisms that may depend on specific cellular conditions and experimental parameters. Based on studies using NO-sensitive dyes, there is evidence that NO production is elevated in cardiomyocytes in response to mechanical stress like cell stretching [5,6]. However, it is still unclear how NO modulates *L*-type Ca^2+^ channels under mechanical stress. Although NO mediates its effects through the soluble guanylyl cyclase/cyclic guanosine monophosphate pathway (sGC/cGMP), others have demonstrated the importance of direct S-nitrosylation of channel proteins [7].

The present investigation aims to reveal the signaling pathways involved in the control of *L*-type Ca^2+^ channels (e.g., Ca_V_1.2) by both mechanical stretch and NO in rat ventricular cardiomyocytes. We particularly focus on the effects of mechanical stretch on channel activity and examine the roles of S-nitrosylation and NO-cGMP pathways in these changes. Understanding these mechanisms is important since they may provide therapeutic targets relevant to cardiac pathologies associated with mechanical stress and changes in Ca^2+^ handling.

Through integrated RNA sequencing, patch-clamp recording, and pharmacological approaches, we show that mechanical stretch uniformly suppressed *I*_Ca,L_ via mechanisms mediated by both pathways. Our findings provide a new perspective on the intricate crosstalk between mechanical stress and NO signaling in the regulation of cardiac Ca^2+^ channels and may open new opportunities for designing targeted therapeutic regimens for heart disease. To gain further insights into the molecular profiles of stretch-induced changes in cardiomyocyte function, we performed genome-wide RNA-seq analysis. The objective was to identify systemic changes in gene expression with a special focus on mechanosensitive ion channels (especially transient receptor potential M7 (TRPM7), Piezo1, and the KCNK family), members of the NO-sGC-cGMP pathway (NOS1–3, GUCY1A3), and enzymes involved in cytoskeletal remodeling and redox homeostasis. Unlike classical qPCR, RNA-seq has the advantage of hypothesis-free scanning for novel transcripts and pathway-level changes that might guide subsequent functional investigations.

## 2. Results

### 2.1. Expression Profile of Voltage-Gated Calcium Channels and Their Auxiliary Subunits

#### 2.1.1. *L*-Type Calcium Channel Predominance in Rat Ventricular Cardiomyocytes

**Figure 1:**
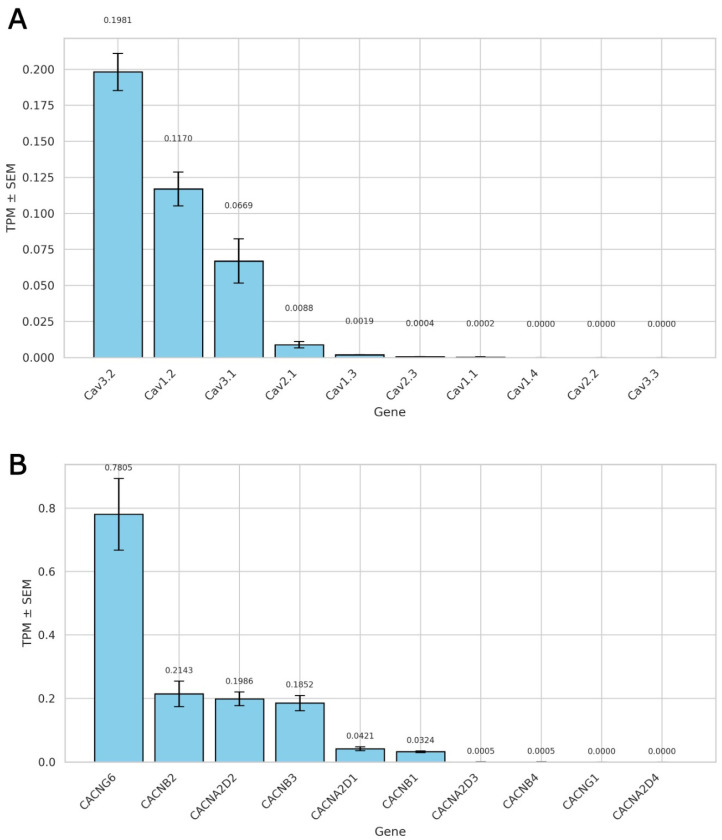
RNA sequencing-based expression profiles of voltage-gated calcium channels and their auxiliary subunits in rat ventricular cardiomyocytes. (**A**) Transcript abundance of pore-forming α1 subunits, expressed as transcripts per million (TPM) ± SEM (*n* = three biological replicates). (**B**) Expression profile of auxiliary subunits associated with *L*-type calcium channels. TPM values ± SEM are shown for three biological replicates.

RNA sequencing of rat ventricular myocytes identified a clear pattern in the expression of voltage-gated calcium channels (Figure 1A). Among the *L*-type channels, Ca_V_1.2 (CACNA1C) was the predominant isoform (0.117 ± 0.0118 TPM), followed by Ca_V_3.2 (CACNA1H) (0.1981 ± 0.0129 TPM) and Ca_V_3.1 (CACNA1G) (0.0669 ± 0.0154 TPM). Ca_V_1.3 (CACNA1D) had minimal expression with 0.0019 ± 0.0002 TPM compared to Ca_V_1.1, Ca_V_1.4, Ca_V_2.2, and Ca_V_3.3, which were undetectable or negligible. Ca_V_2.1 (0.0088 ± 0.0023 TPM) and Ca_V_2.3 (0.0004 ± 0.0002 TPM) were found at very low levels, as expected, as they are mainly expressed in neuronal tissues.

These data from three independent biological replicates unambiguously identify Ca_V_1.2 as the predominant voltage-gated calcium channel in adult rat ventricular cardiomyocytes.

#### 2.1.2. Auxiliary Subunit Expression Profile of *L*-Type Calcium Channels

To fully characterize the molecular composition of *L*-type calcium channel complexes in rat ventricular myocytes, we determined the expression profile of auxiliary subunits α2δ and β that are known to alter channel function (Figure 1B). Among β subunits, CACNB2 (0.2143 ± 0.0400 TPM) was most abundant, followed by CACNB3 (0.1852 ± 0.0239 TPM) and CACNB1 (0.0324 ± 0.0025 TPM), with very low levels of CACNB4 (0.0005 ± 0.0001 TPM). Regarding the α2δ subunits, CACNA2D2 (0.1986 ± 0.0219 TPM) and CACNA2D1 (0.0421 ± 0.0062 TPM) were predominant, whereas CACNA2D3 and CACNA2D4 were expressed below the detection limit. Among γ subunits, only CACNG6 showed substantial expression (0.7805 ± 0.1128 TPM) while CACNG1 was not detected. These auxiliary subunits likely contribute to the functional regulation of Ca_V_1.2 channel complexes in the ventricular myocardium. Notably, the majority of γ subunits (CACNG2–8) were excluded from this analysis, as they have been functionally reclassified as transmembrane AMPA receptor regulatory proteins (TARPs) rather than true auxiliary calcium channel subunits [8,9]. No members of the γ family have been recognized as bona fide *L*-type calcium channel modulators except CACNG1 and CACNG6 [10,11].

Collectively, these data indicate that CACNB2, CACNB3, CACNA2D2, CACNA2D1, and CACNG6 are the predominant auxiliary subunits expressed in rat ventricular cardiomyocytes. The substantial expression of these regulators serves as a molecular blueprint for the regulation of Ca_V_1.2 function in subsequent electrophysiological studies.

#### 2.1.3. Implications for *T*-Type Channel Contribution to Recorded Currents

Although *T*-type calcium channels—particularly Ca_V_3.1—were detectably expressed in the RNA sequencing dataset used in our experiment, their functional role in the recorded calcium currents was insignificant due to the specific design of our electrophysiological protocol. Patch-clamp recordings were performed using a *V*_h_ of −80 mV, a holding potential that effectively inactivates *T*-type Ca^2+^ channels—typically activated at more negative potentials (approximately −70 to −40 mV). Furthermore, our pulse protocol, which ranged from −40 mV to +40 mV, was focused on a voltage range that preferentially activates *L*-type Ca^2+^ channels, ensuring that the recorded currents predominantly reflected *L*-type activity. In addition, the absence of mechanosensitivity in *T*-type channels is supported by the literature. Foundational electrophysiology has shown that *T*-type channels do not open in response to mechanical stimuli (hydrostatic pressure, membrane stretch) that robustly open channels of other types, such as *N*-type channels [12,13]. This functional separation is consistent with structural studies that have shown that *T*-type channels do not have the important mechanosensitive motifs (including amphipathic helices and lipid-sensitive domains) present in many stretch-activated ion channels [14].

All of these points together support that the stretch-modulated Ca^2+^ currents we observed in our experiments mainly represent *L*-type Ca_V_1.2 channels as the predominant functional subtype in rat ventricular cardiomyocytes, which are structurally suitable for mechanotransduction.

#### 2.1.4. Impact of Mechanical Stretch on *L*-Type Ca^2+^ Currents

**Figure 2:**
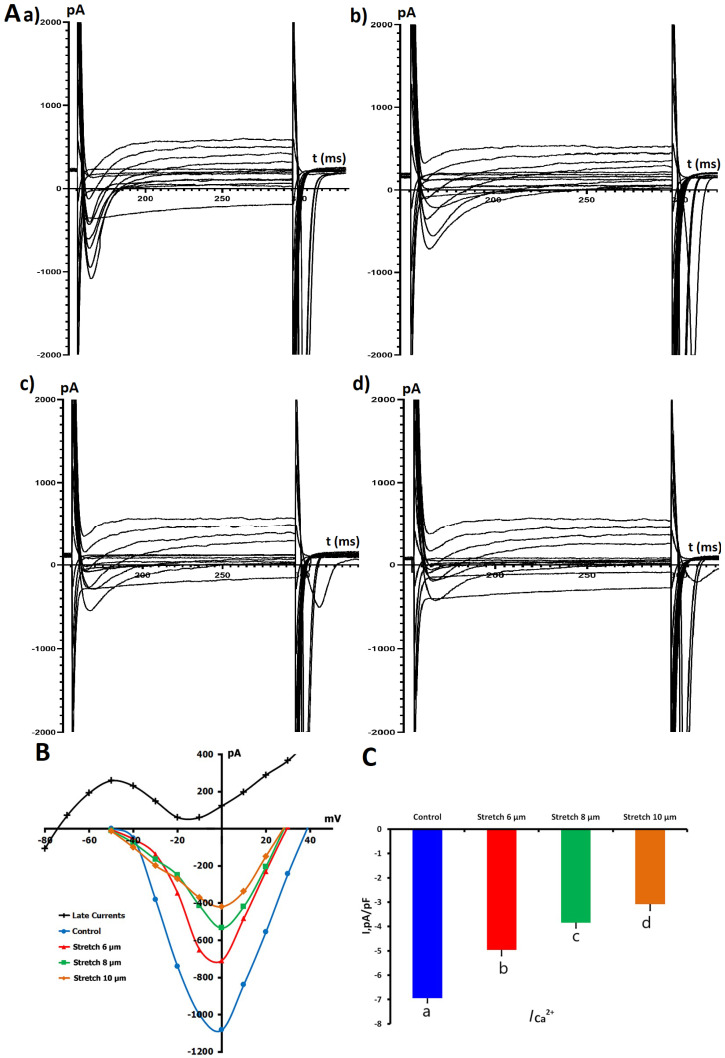
Effects of mechanical stretch on *L*-type Ca^2+^ current (*I*_Ca,L_) in rat ventricular cardiomyocytes. (**A**) Representative whole-cell *I*_Ca,L_ traces recorded under control conditions (**a**) and during mechanical stretch of 6 μm (**b**), 8 μm (**c**), and 10 μm (**d**). Currents were elicited using a standard depolarizing pulse protocol (for details, please see the Materials and Methods section). (**B**) Current–voltage (*I*/*V*) relationships of *I*_Ca,L_ under control conditions and during mechanical stretch of 6, 8, and 10 μm. The net late current (*I*_L,Net_; for clarification, please see the Materials and Methods section) is shown in black, indicating time-dependent decay during sustained depolarization. (**C**) Mean peak *I*_Ca,L_ current density (pA/pF) under control conditions and at increasing levels of stretch (6, 8, and 10 μm). Lowercase letters (a, b, c, d) above the bars indicate statistically significant differences between the groups (*p* < 0.05). Statistical comparisons were made using repeated measures ANOVA with the Holm–Sidak post hoc test. The data are presented as mean ± SEM. Superimposed traces in panels (**A**(**a**–**d**)) partially overlap at later time points due to the nature of multi-trace electrophysiological recordings. This overlap does not affect the interpretation of current amplitude, kinetics, or time scale.

We investigated the mechanosensitivity of *I*_Ca,L_ through measurement of *I*_Ca,L_ before and during applied axial stretch. To maintain high-quality electrophysiological data, only cells with stable access resistance and <10% baseline current drift over the course of the protocol were included for analysis. In Figure 2, the voltage-dependent properties of *I*_Ca,L_ and the modulation of *I*_Ca,L_ by different amounts of mechanical stretch (6, 8, and 10 μm) are shown.

Under control conditions, in K^+^_in_/K^+^_out_ solution, baseline *I*_Ca,L_ density was −6.95 ± 0.18 pA/pF (*n* = 7, *m* = 4), with sarcomere length 1.83 ± 0.01 μm. Local axial stretch produced a consistent and graded reduction in *I*_Ca,L_ amplitude, correlating with increases in sarcomere length. *I*_Ca,L_ reduction was observed with 6 μm stretch (sarcomere length 2.00 ± 0.01 μm), where *I*_Ca,L_ diminished to −4.96 ± 0.24 pA/pF. The current continued to decrease with 8 μm stretch (sarcomere length 2.10 ± 0.01 μm) to −3.84 ± 0.22 pA/pF, reaching −3.08 ± 0.19 pA/pF at 10 μm stretch (sarcomere length 2.17 ± 0.01 μm). All changes were statistically significant compared to the control (*p* < 0.01).

#### 2.1.5. Effects of NO Donor SNAP on *I*_Ca,L_

**Figure 3:**
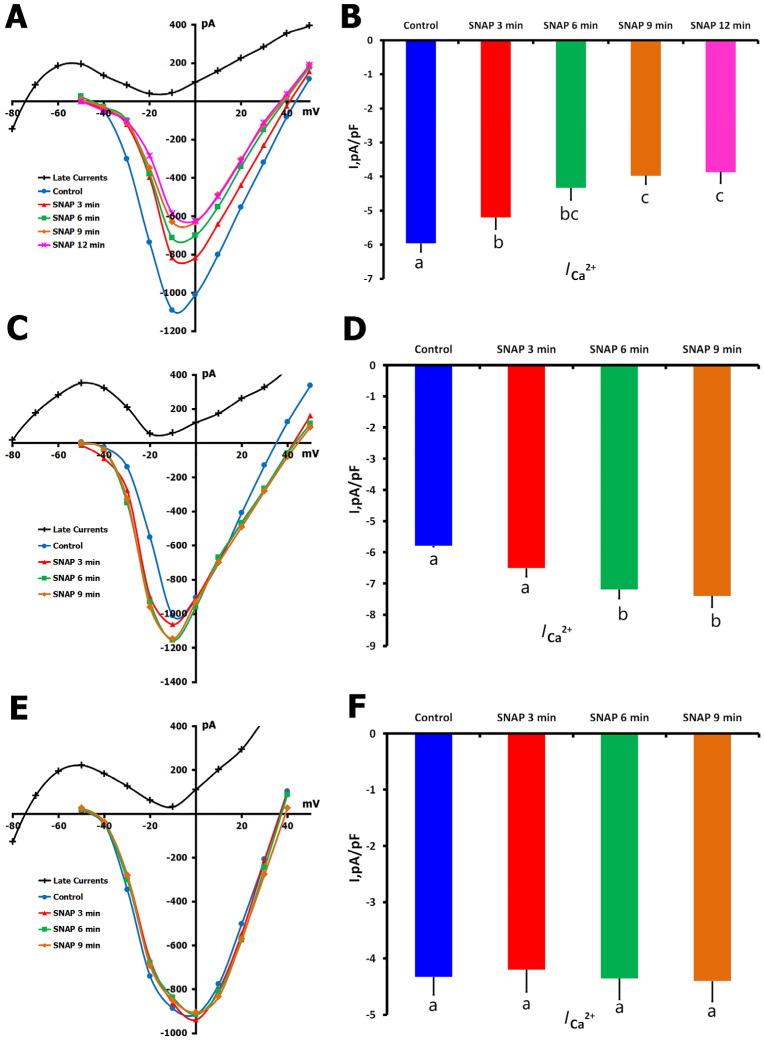
Effects of SNAP (200 µmol/L) on *I*_Ca,L_ in unstretched cells in K^+^_in_/K^+^_out_ solutions: (reduction in *I*_Ca,L_ (**A**,**B**), enhancement of *I*_Ca,L_ (**C**,**D**), and no significant current size change (**E**,**F**). (**A**) Representative *I*/*V* curves under control conditions and following 3, 6, 9, and 12 min of SNAP application. Cell capacitance = 175 pF. (**B**) Mean *I*_Ca,L_ density at baseline and after 3, 6, 9, and 12 min of SNAP exposure. Lowercase letters (a, b, c) above bars indicate statistically significant differences between the time points (*p* < 0.05). (**C**) *I*/*V* curves showing *I*_Ca,L_ under control conditions and at 3, 6, and 9 min after SNAP application. Cell capacitance = 170 pF. (**D**) Mean *I*_Ca,L_ density at baseline and during SNAP exposure. Lowercase letters (a, b) denote significant differences between groups (*p* < 0.05). (**E**) *I*/*V* curves recorded under control conditions and following 3, 6, and 9 min of SNAP application. Cell capacitance = 120 pF. (**F**) Mean *I*_Ca,L_ density remained unchanged over time (*p* = ns across all time points). Lowercase letters (a) indicate no statistically significant differences between groups. For all *I*/*V* plots (**A**,**C**,**E**), the net late current (*I*_L,Net_) used for *I*_Ca,L_ calculation is depicted in black. Statistical comparisons were made using repeated measures ANOVA with the Holm–Sidak post hoc test. The data are presented as mean ± SEM.

Heterogeneities in the modulation of *I*_Ca,L_ by application of S-nitroso-N-acetyl-D,L-penicillamine (SNAP) (200 µM) in unstretched cardiomyocytes were obtained, revealing three distinct response profiles. These are shown in Figure 3 as a reduction in *I*_Ca,L_ (Figure 3A,B), an enhancement of *I*_Ca,L_ (Figure 3C,D), and no significant current size change (Figure 3E,F).

The most common response (66.6% of cells; *n* = 22) was an *I*_Ca,L_ decrease from −5.96 ± 0.25 to −3.87 ± 0.33 pA/pF after 12 min of SNAP exposure. During the treatment phase, a statistically significant difference compared to the control was found, but no significant differences were confirmed between time points (3, 6, 9, and 12 min; *p* = ns).

In a smaller subset of cells (9%; *n* = 3), SNAP significantly enhanced *I*_Ca,L_ from −5.79 ± 0.31 to −7.40 ± 0.36 pA/pF after 9 min (*p* < 0.01 vs. control), with no significant differences between intermediate time points (*p* = ns).

The remaining cells (24.4%, *n* = 8) showed no change in *I*_Ca,L_ throughout the recording period, as compared with the baseline (control: −7.72 ± 0.23 pA/pF; *p* = ns at each time point).

#### 2.1.6. Effects of SNAP on *I*_Ca,L_ During Mechanical Stretch

To address the interplay between NO signaling and mechanical stress in the modulation of *L*-type Ca^2+^ channels, we applied the following two complementary experimental protocols:

(1) SNAP application during sustained mechanical stretch;
(2) Mechanical stretch was applied to cells pretreated with SNAP.

**Figure 4:**
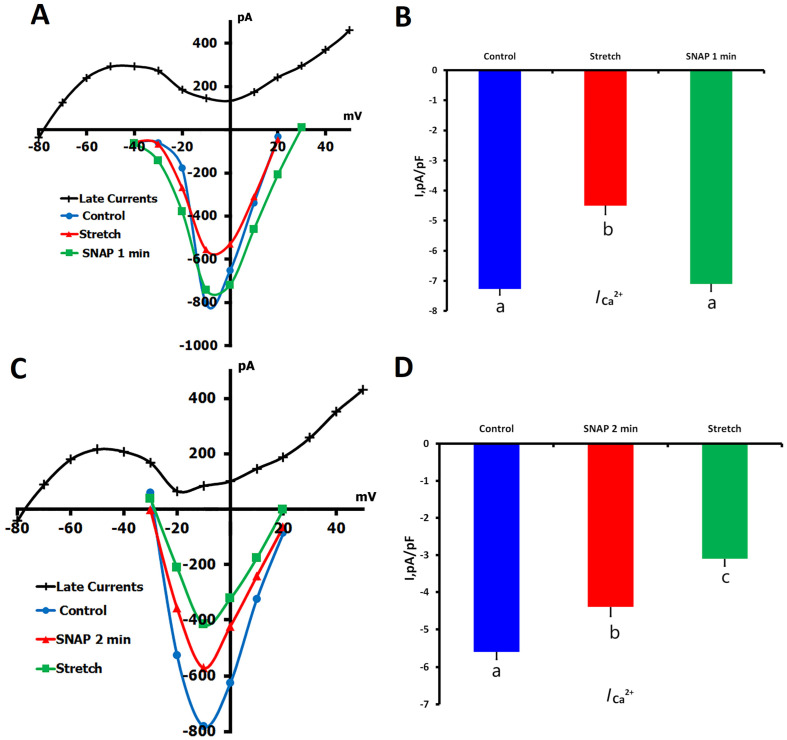
Effects of mechanical stretch and SNAP application on *I*_Ca,L_ in rat ventricular cardiomyocytes. Experiments were performed in K^+^_in_/K^+^_out_ solutions to evaluate how SNAP (200 µM) modulates *I*_Ca,L_ during and after mechanical stretch. (**A**) Representative *I*/*V* curves show *I*_Ca,L_ under control conditions (blue circles), during 6 μm axial stretch (red triangles), and after 1 min of SNAP application while stretch was maintained (green squares). Cell capacitance = 135 pF. (**B**) Mean *I*_Ca,L_ densities corresponding to each condition. Lowercase letters (a, b) above the bars indicate statistically significant differences between the groups (*p* < 0.05). (**C**) *I*/*V* curves demonstrate *I*_Ca,L_ under control conditions (blue circles), after 2 min of SNAP application (red triangles), and during subsequent 6 μm stretch (green squares). Cell capacitance = 150 pF. (**D**) Mean *I*_Ca,L_ densities for each condition. Lowercase letters (a, b, c) above the bars indicate statistically significant differences between the groups (*p* < 0.05). For both (**A**,**C**), the net late current (*I*_L,Net_) used to calculate *I*_Ca,L_ is shown as a black curve. Statistical comparisons were made using repeated measures ANOVA with the Holm–Sidak post hoc test. Data are presented as mean ± SEM.

In protocol 1, application of local axial stretch (6 μm) significantly decreased *I*_Ca,L_ from −7.27 ± 0.20 pA/pF (control) to −4.50 ± 0.30 pA/pF (*p* < 0.01; *n* = 7, *m* = 5). When SNAP (200 μM) was perfused during the maintained stretch, *I*_Ca,L_ quickly returned to −7.10 ± 0.40 pA/pF within 1 min (*p* = ns vs. control), a value not significantly different from the baseline, but significantly higher than during stretching alone (*p* < 0.01). These results indicate that NO can rapidly nullify the effect of stretch on *L*-type Ca^2+^ channels (Figure 4A,B).

In the second protocol, SNAP was applied to unstretched cells, followed by a decrease in *I*_Ca,L_ from −5.60 ± 0.40 pA/pF to −4.50 ± 0.40 pA/pF after 2 min (*p* < 0.01). When these SNAP-treated cells were subsequently subjected to 6 μm stretch, *I*_Ca,L_ was further reduced to −3.30 ± 0.40 pA/pF (*p* < 0.01 vs. both control and SNAP alone), demonstrating that the mechanical stimulus was able to further reduce basal myocyte *I*_Ca,L_ when superimposed on that from SNAP (Figure 4C,D).

The divergent results observed in these two experimental settings—SNAP reversing stretch-induced inhibition when applied after stretch but augmenting inhibition when applied before stretch—serve to illustrate the temporal context of both the NO and the mechanical pathways. These findings are consistent with the idea that NO and stretch regulate *I*_Ca,L_ via partly independent but convergent mechanisms and that the particular sequence of activation is crucial for dictating channel behavior. The rapid reversibility of stretch effects by SNAP also indicates a dynamic regulatory mechanism that may be pertinent to potential therapy for diseases associated with altered mechanical load (cardiac hypertrophy or failure).

### 2.2. Effects of sGC Inhibition by ODQ on I_Ca,L_ Regulation

#### 2.2.1. ODQ Inhibition of *I*_Ca,L_ in Unstretched Cells

**Figure 5:**
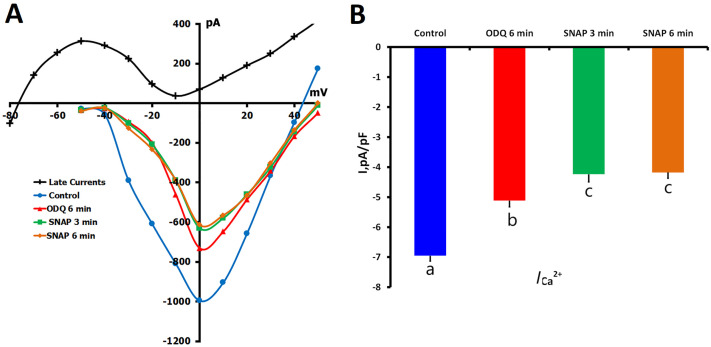
Effects of ODQ (5 µM) and SNAP (200 µM) on *I*_Ca,L_ in unstretched rat ventricular cardiomyocytes. Experiments were conducted in K^+^_in_/K^+^_out_ solutions to assess the influence of soluble guanylyl cyclase (sGC) inhibition and subsequent NO donor application on *I*_Ca,L_. (**A**) Representative *I*/*V* curves show *I*_Ca,L_ under control conditions (blue circles), after 6 min of ODQ application (red triangles), and following SNAP addition at 3 min (green squares) and 6 min (orange diamonds). The late component of the current (*I*_L,Net_), used for current density calculation, is shown in black. Cell capacitance = 155 pF. (**B**) Mean *I*_Ca,L_ densities for each condition. Lowercase letters (a, b, c) above the bars indicate statistically significant differences between the groups (*p* < 0.05). Statistical comparisons were made using repeated measures ANOVA with the Holm–Sidak post hoc test. Data are presented as mean ± SEM.

To determine the involvement of the NO-sGC-cGMP signaling pathway in the regulation of *L*-type Ca^2+^ channels, we initially investigated the impact of sGC inhibition by 1H-[1,2,4]-oxadiazolo[4,3-a]quinoxalin-1-one (ODQ) (5 μM) in unstretched ventricular myocytes. *I*_Ca,L_ was significantly attenuated by the application of ODQ for 6 min from −6.88 ± 0.18 pA/pF to −5.23 ± 0.21 pA/pF (*p* < 0.01; *n* = 13, *m* = 7), suggesting that tonic sGC activity is involved in determining basal Ca^2+^ current amplitude (Figure 5A,B).

Upon subsequent introduction of SNAP (200 μM) into the ODQ-containing perfusate *I*_Ca,L_ decreased further to −4.49 ± 0.25 pA/pF at 3 min (*p* < 0.01 vs. ODQ alone) with no further significant change at 6 min (−4.45 ± 0.19 pA/pF; *p* = ns vs. 3-min value). This additive inhibition indicates that SNAP affects *I*_Ca,L_ through sGC-independent pathways, probably through other NO signaling pathways such as S-nitrosylation.

#### 2.2.2. ODQ Effects on *I*_Ca,L_ During Mechanical Stretch

**Figure 6:**
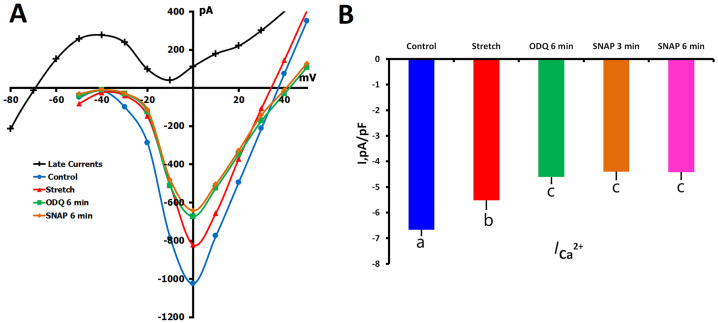
Combined effects of mechanical stretch, ODQ (5 µM), and SNAP (200 µM) on *I*_Ca,L_ in rat ventricular cardiomyocytes. Experiments were performed in K^+^_in_/K^+^_out_ solutions to assess how sGC inhibition and NO signaling interact with mechanical stress to regulate *I*_Ca,L_. (**A**) Representative *I*/*V* curves show *I*_Ca,L_ under control conditions (blue circles), during local axial stretch of 6 μm (red triangles), and after 6 min of ODQ application while stretch was maintained (green squares). The net late current (*I*_L,Net_), used for current density calculation, is shown in black. Cell capacitance = 170 pF. (**B**) Mean *I*_Ca,L_ densities under each condition. Lowercase letters (a, b, c) above the bars indicate statistically significant differences between the groups (*p* < 0.05). Statistical analysis was performed using repeated measures ANOVA with the Holm–Sidak post hoc test. Data are presented as mean ± SEM.

We further investigated the effect of sGC inhibition on the regulation of Ca^2+^ current under mechanical stress. In these experiments (*n* = 7, *m* = 4), 6 μm mechanical stretch decreased *I*_Ca,L_ from control levels of −6.67 ± 0.22 pA/pF to −5.52 ± 0.35 pA/pF (*p* < 0.01), as previously reported (Figure 6A,B). ODQ applied subsequently reduced *I*_Ca,L_ to −4.61 ± 0.26 pA/pF after 6 min (*p* < 0.01 vs. stretch alone).

In the case of stretched myocytes, the presence of SNAP did not produce notable modifications of *I*_Ca,L_ when the cells were subjected to ODQ: −4.42 ± 0.40 pA/pF at 3 min and −4.48 ± 0.42 pA/pF at 6 min (*p* = ns). This insensitivity toward SNAP is in contrast to the further inhibitory effect in unstretched cells, implying that mechanical stretch modulates the channel sensitivity to NO-mediated regulation.

Collectively, the findings under stretched and unstretched conditions suggest that the NO-sGC-cGMP pathway participates in the modulation of Ca^2+^ channels in a context-specific manner. Mechanical stress also seems to alter the balance of sGC-dependent and sGC-independent NO signaling, indicating the dynamic crosstalk between mechano-stimulated and biochemical control of *L*-type Ca^2+^ channels in cardiomyocytes.

### 2.3. Effects of Ascorbic Acid (AA) on I_Ca,L_ Regulation

#### 2.3.1. AA Modulation of *I*_Ca,L_ in Unstretched Cells

To examine the role of S-nitrosylation in the modulation of *L*-type Ca^2+^ channels, we used ascorbic acid (AA, 10 μM), a reducing agent that has been shown to specifically remove S-nitrosylation from proteins. In our study, the effect of AA alone and its interaction with NO donor SNAP in unstretched ventricular cardiomyocytes were examined.

**Figure 7:**
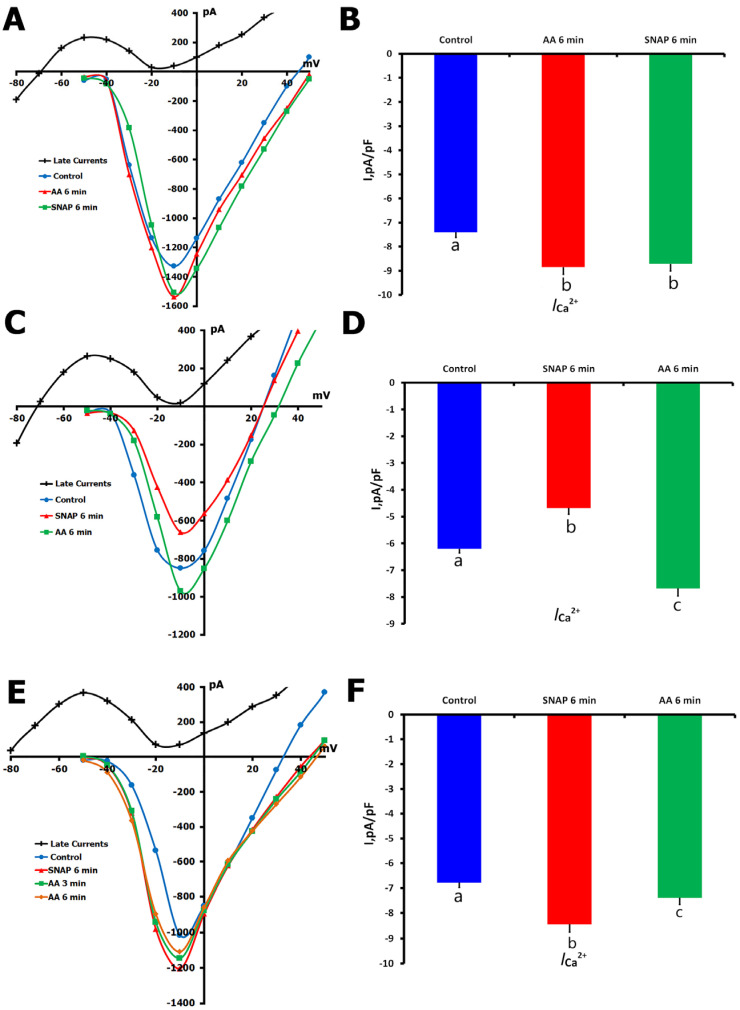
Effects of ascorbic acid (AA, 10 µM) and SNAP (200 µM) on *I*_Ca,L_ in unstretched rat ventricular cardiomyocytes. Experiments were performed in K^+^_in_/K^+^_out_ solutions to evaluate how AA modulates *I*_Ca,L_ under basal conditions and in the presence of the NO donor SNAP. (**A**) Representative *I*/*V* curves showing *I*_Ca,L_ under control conditions (blue circles), after 6 min of AA perfusion (red triangles), and following an additional 6 min of SNAP application in the presence of AA (green squares). The net late current (*I*_L,Net_) is shown in black; cell capacitance = 180 pF. (**B**) Mean *I*_Ca,L_ values under the same conditions. Lowercase letters (a, b) above the bars indicate statistically significant differences between the groups (*p* < 0.05). (**C**) *I*/*V* curves show *I*_Ca,L_ under control conditions (blue circles), after 6 min of SNAP application (red triangles), and following 6 min of AA perfusion (green squares); cell capacitance = 135 pF. (**D**) Mean *I*_Ca,L_ values under the same conditions. Lowercase letters (a, b, c) above the bars indicate statistically significant differences between the groups (*p* < 0.05). (**E**) *I*/*V* curves show *I*_Ca,L_ under control conditions (blue circles), after 6 min of SNAP application (red triangles), and following an additional 6 min of AA application (green squares); cell capacitance = 155 pF. (**F**) Mean *I*_Ca,L_ values under the same conditions. Lowercase letters (a, b, c) above the bars indicate statistically significant differences between the groups (*p* < 0.05). In all panels (**A**,**C**,**E**), *I*_L,Net_ used for current density calculation is shown in black. Statistical analysis was performed using repeated measures ANOVA with the Holm–Sidak post hoc test. Data are presented as mean ± SEM.

In the first series of experiments (*n* = 8, *m* = 3), we tested the impact of AA application alone. Baseline *I*_Ca,L_ was −7.57 ± 0.02 pA/pF and increased to −8.77 ± 0.03 pA/pF after 6 min of AA perfusion (*p* < 0.01; Figure 7A,B). When 200 μM SNAP was added in the continued presence of AA, there was no significant change in *I*_Ca,L_ (−8.54 ± 0.03 pA/pF at 6 min, *p* = ns compared to AA alone). This indicates that AA pretreatment prevented SNAP-induced channel modulation, possibly by scavenging S-nitrosylation targets before the addition of SNAP.

In a second series of experiments, we reversed the order of application to assess the effect of AA on SNAP-modulated *I*_Ca,L_. We identified two distinct response patterns.

In the first pattern (*n* = 7, *m* = 4), SNAP alone decreased *I*_Ca,L_ from −6.44 ± 0.15 pA/pF to −5.02 ± 0.23 pA/pF over 6 min (*p* < 0.01). Subsequent AA application reversed *I*_Ca,L_ to −7.37 ± 0.28 pA/pF (*p* < 0.01 versus SNAP; Figure 7C,D), which demonstrated that AA successfully reversed the SNAP-induced suppression, supporting the major role of S-nitrosylation in the inhibitory effect.

In the second pattern (*n* = 6, *m* = 3), SNAP first increased *I*_Ca,L_ from −7.10 ± 0.02 pA/pF to −8.25 ± 0.31 pA/pF (*p* < 0.01). The enhancement of *I*_Ca,L_ by SNAP was partially reversed by AA treatment, −7.59 ± 0.03 pA/pF (*p* < 0.01 vs. SNAP; Figure 7E,F), but the resultant amplitude was still significantly greater than that of control (*p* < 0.05). This indicates that SNAP’s stimulatory effect is mediated by S-nitrosylation as well as by a non-S-nitrosylation mechanism.

Taken together, these findings underscore the key role of S-nitrosylation in the regulation of *L*-type Ca^2+^ channel activity and uncover the dual potential of NO signaling that is considered in light of the redox status of the channels.

#### 2.3.2. AA Effects on Stretch-Reduced *I*_Ca,L_

**Figure 8:**
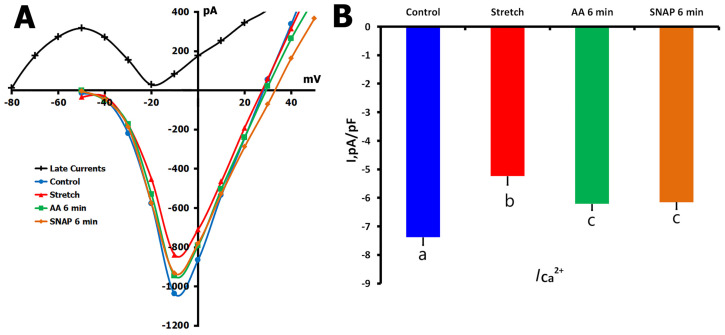
Effects of ascorbic acid (AA, 10 µM) and SNAP (200 µM) on *I*_Ca,L_ in mechanically stretched rat ventricular cardiomyocytes. Experiments were conducted in K^+^_in_/K^+^_out_ solutions to assess whether AA and SNAP modulate stretch-induced suppression of *I*_Ca,L_. (**A**) Representative *I*/*V* curves show *I*_Ca,L_ under control conditions (blue circles), during 6 μm axial stretch (red triangles), and after 6 min of AA perfusion while stretch was maintained (green squares). Subsequent SNAP application for 6 min produced no additional significant change (orange diamonds). The late current (*I*_L,Net_) used for *I*_Ca,L_ calculation is shown in black; cell capacitance = 170 pF. (**B**) Mean *I*_Ca,L_ densities across all conditions. Lowercase letters (a, b, c) above the bars indicate statistically significant differences between the groups (*p* < 0.05). Statistical analysis was performed using repeated measures ANOVA with the Holm–Sidak post hoc test. Data are presented as mean ± SEM.

Following the characterization of AA’s effects on basal Ca^2+^ channel activity, we next investigated its ability to modulate *I*_Ca,L_ in cardiomyocytes subjected to mechanical stretch. In these experiments (*n* = 7, *m* = 3), mechanical stretching of the cell membrane by 6 μm significantly reduced *I*_Ca,L_ from a control value of −7.31 ± 0.28 pA/pF to −5.24 ± 0.31 pA/pF (*p* < 0.01; Figure 8A,B).

Perfusion with AA (10 μM) for 6 min partially restored the Ca^2+^ current, resulting in an *I*_Ca,L_ of −6.11 ± 0.22 pA/pF (*p* < 0.01 vs. stretch; *p* < 0.05 vs. control), indicating that at least part of the stretch-induced inhibition is mediated through S-nitrosylation of *L*-type Ca^2+^ channels. In contrast, in the continued presence of mechanical stretch, when SNAP (200 μM) was added after AA treatment, no further reduction of *I*_Ca,L_ was observed (−6.05 ± 0.25 pA/pF, *p* = ns vs. AA alone), suggesting no further regulation of *I*_Ca,L_ by NO at this time point.

These results suggest that mechanical stretch decreases *I*_Ca,L_, at least in part, through pathways related to S-nitrosylation, and that AA can reverse this effect. However, the partial recovery and its resistance to additional SNAP modulation indicate the involvement of S-nitrosylation-independent mechanisms in stretch-induced downregulation of Ca^2+^ channel activity.

### 2.4. Effects of NEM on I_Ca,L_ Regulation

#### 2.4.1. Biphasic NEM Effects on *I*_Ca,L_ in Unstretched Cells

To further investigate the role of protein thiol groups in Ca^2+^ channel regulation, we employed N-ethylmaleimide (NEM), which irreversibly alkylates thiol groups and prevents S-nitrosylation. Due to the compound’s cellular toxicity at physiological temperature, these experiments were conducted at 22 °C with careful monitoring of cell viability. Initial experiments revealed that even at reduced temperatures, NEM concentrations above 200 μM or extended exposure time led to rapid cell deterioration, consistent with previous reports in the literature.

**Figure 9:**
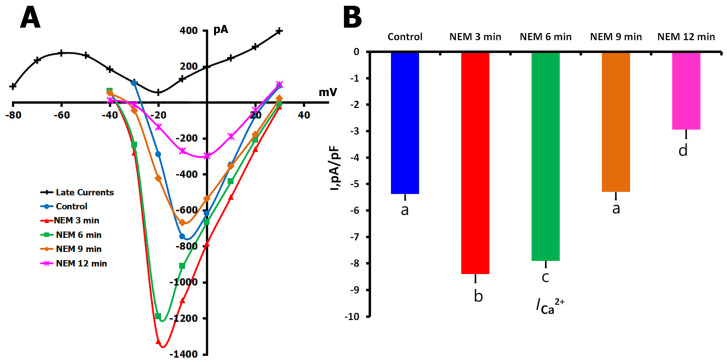
Biphasic effects of N-ethylmaleimide (NEM, 200 µM) on *I*_Ca,L_ in unstretched rat ventricular cardiomyocytes at 22 °C. Experiments were conducted in K^+^_in_/K^+^_out_ solutions to evaluate the time-dependent effects of thiol alkylation by NEM on *I*_Ca,L_ under basal (unstretched) conditions. (**A**) Representative *I*/*V* curves show *I*_Ca,L_ at baseline (blue circles) and following 3 min (red triangles), 6 min (green squares), 9 min (orange diamonds), and 12 min (purple stars) of continuous NEM perfusion. The net late current (*I*_L,Net_) used for current calculation is shown in black. Cell capacitance = 160 pF. (**B**) Mean *I*_Ca,L_ densities corresponding to each time point. Lowercase letters (a, b, c, d) above the bars indicate statistically significant differences between the groups (*p* < 0.05). Statistical comparisons were made using repeated measures ANOVA with the Holm–Sidak post hoc test. Data are presented as mean ± SEM.

In unstretched cells (*n* = 10, *m* = 4), NEM (200 μM) produced a striking biphasic response in *I*_Ca,L_ (Figure 9A,B). From control values of −5.40 ± 0.3 pA/pF, the current increased dramatically to −8.40 ± 0.3 pA/pF within the first 3 min of NEM exposure. This initial increase was followed by a slight decline to −7.90 ± 0.4 pA/pF at 6 min, then a more substantial reduction to near-control levels (−5.30 ± 0.3 pA/pF) at 9 min. By 12 min, *I*_Ca,L_ had fallen significantly below control values to −2.92 ± 0.2 pA/pF. This complex temporal response pattern suggests that different populations of thiol groups, possibly with varying accessibility or reactivity, contribute to Ca^2+^ channel regulation.

#### 2.4.2. NEM Effects on Stretch-Modified *I*_Ca,L_

**Figure 10:**
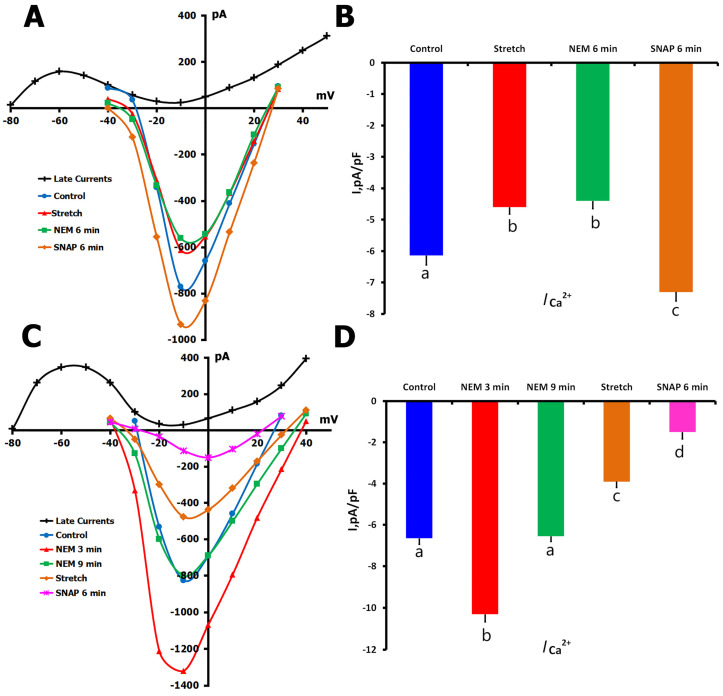
Effects of N-ethylmaleimide (NEM, 200 µM) and SNAP (200 µM) on *I*_Ca,L_ during mechanical stretch in rat ventricular cardiomyocytes at 22 °C. Experiments were performed in K^+^_in_/K^+^_out_ solutions to evaluate how thiol alkylation by NEM and NO signaling via SNAP affect *I*_Ca,L_ under conditions of mechanical stress. (**A**) Representative *I*/*V* curves show *I*_Ca,L_ under control conditions (blue circles), after 6 μm axial stretch (red triangles), and following 6 min of NEM perfusion during stretch (green squares). The net late current (*I*_L,Net_) used for current calculation is shown in black. Cell capacitance = 130 pF. (**B**) Mean *I*_Ca,L_ densities under the same conditions. Lowercase letters (a, b, c) above the bars indicate statistically significant differences between the groups (*p* < 0.05). (**C**) *I*/*V* curves show *I*_Ca,L_ under control conditions (blue circles), after 3 min of NEM perfusion (red triangles), and after an additional 6 min of NEM (green squares). *I*_L,Net_ is shown as a black curve; cell capacitance = 130 pF. (**D**) Mean *I*_Ca,L_ densities for each condition. Lowercase letters (a, b, c, d) above the bars indicate statistically significant differences between the groups (*p* < 0.05). Statistical comparisons were made using repeated measures ANOVA with the Holm–Sidak post hoc test. Data are presented as mean ± SEM.

To understand how thiol modification affects stretch-dependent channel regulation, we employed two distinct experimental protocols (Figure 10). In the first approach (*n* = 7, *m* = 3), we initially stretched the cells, confirming the typical reduction in *I*_Ca,L_ from −6.14 ± 0.3 pA/pF to −4.60 ± 0.22 pA/pF (Figure 10A,B). Subsequent application of NEM to these stretched cells produced minimal change in current amplitude (−4.40 ± 0.29 pA/pF). However, when SNAP was added to the NEM-containing solution, we observed a surprising increase in *I*_Ca,L_ to −7.30 ± 0.33 pA/pF, exceeding control levels.

In the second protocol (*n* = 6, *m* = 3), we reversed the sequence by applying NEM before mechanical stretch (Figure 10C,D). The initial NEM application produced the characteristic biphasic response, with *I*_Ca,L_ increasing from −6.65 ± 0.29 pA/pF to −10.3 ± 0.37 pA/pF after 3 min, then returning to near-control levels (−6.55 ± 0.25 pA/pF) after 12 min. When these NEM-treated cells were subsequently stretched, *I*_Ca,L_ decreased to −3.91 ± 0.29 pA/pF, and the addition of SNAP caused a further reduction to −1.66 ± 0.33 pA/pF.

The contrasting responses to SNAP in these two protocols—enhancement of current when applied after stretch versus inhibition when applied before stretch—suggest that the temporal sequence of thiol modification and mechanical stress critically determines the final functional state of the channels. Furthermore, the ability of mechanical stretch to modify channel function even after NEM treatment indicates that some aspects of stretch-dependent regulation may occur independently of thiol group availability.

## 3. Discussion

### 3.1. Expression Pattern and Mechanosensitivity

Our RNA-seq data conclusively validate Ca_V_1.2 (CACNA1C) as the dominant member of the group of voltage-gated calcium channels expressed in rat ventricular cardiomyocytes, both in overall transcript amount and distribution among other *L*- and *T*-type isoforms [15,16]. Notably, Ca_V_1.3 and Ca_V_1.1 had negligible expression, while *T*-type channels (particularly Ca_V_3.1 and Ca_V_3.2) were expressed at magnitudes far below those of Ca_V_1.2. Functionally, this expression profile is highly supportive of our conclusion that *I*_Ca,L_ recorded in our studies is mostly due to Ca_V_1.2 channel activity with minimal contribution from T-type calcium channels. In addition, their auxiliary subunit profile (with high levels of CACNB2, CACNA2D2, and CACNG6 expression) is indicative of a molecular composition more suitable for greater functional modulation of Ca_V_1.2. Critically, these findings provide a molecular rationale for our decision to narrow our investigations of mechanosensitivity and NO modulation to Ca_V_1.2 channel activity and exclude significant confounding effects of other Ca^2+^ channel subtypes.

We found a uniform suppression of *I*_Ca,L_ during mechanical stretch, which supported previous results in different preparations [17,18]. Similar Ca^2+^ current reductions in response to axial stretch have been found in guinea pig ventricular myocytes [17,18], indicating a common mechanosensitive mechanism among species. F-actin or similar cytoskeletal components, e.g., microtubules, have also been suggested to participate in the modulation of mechanosensitive channels and Ca_V_1.2 gating, providing additional evidence for an integrated structural–functional response to mechanical stimulation [19].

Although some heterogeneity was observed in *I*/*V* relationships, it may be attributed to the intrinsic cell-to-cell variability in cells isolated from the tissue. To ensure data reliability, only recordings with stable series resistance and comparable membrane capacitance were included. Representative current traces also confirm the reproducibility of our electrophysiological findings (Figure 2A).

Collectively, the RNA-seq data confirm Ca_V_1.2 as the major *L*-type Ca^2+^ channel isoform in this cellular environment, thus rationalizing the observed electrophysiological effects (Figure 1A,B). The stretch-dependent decrease in *I*_Ca,L_ and its modulation by NO donors and inhibitors reflect changes in the functional state of Ca_V_1.2 activity. Interestingly, NO signaling seems able to suppress this mechanosensitive suppression and even promote it. This dual sensitivity is probably a consequence of separate yet overlapping mechanisms (i.e., S-nitrosylation and cGMP-mediated signaling) that work autonomously but interact with mechanical stimuli. These findings underscore the significance of temporal dynamics and subcellular compartmentalization in deciphering the manner by which mechanical- and redox-activated signals converge at the level of a single, biologically relevant ion channel.

### 3.2. NO Signaling Complexity and Mechanisms of Regulation

The differential modulation of Ca^2+^ channels by NO underscores the complex control of cardiac electrophysiological properties by redox signaling. Previous studies have shown conflicting results for the effect of NO donors: SNAP increased basal *I*_Ca,L_ in human atrial myocytes [20], while it decreased the current in neonatal rat ventricular strips [21]. These data are in accordance with our observation that biphasic changes induced by nitro-L-arginine methyl ester (L-NAME) or diethylenetriaminepentaacetic acid (DTPA) were seen, which probably corresponded to drug concentrations, as has been reported with 3-morpholinosydnonimine (SIN-1) in frog ventricular myocytes [22].

Such apparent inconsistencies can be explained by multiple levels of physiological complexity. The first is due to the fact that the three isoforms of NO synthase (NOS)—neuronal (nNOS), endothelial (eNOS), and inducible (iNOS)—have different subcellular localizations and activation pathways. For instance, eNOS is membrane-bound and can be stimulated mechanically, while nNOS is located near the sarcoplasmic reticulum and transversal tubules [23]. These spatial differences lead to an NO production that is locally regulated and may affect the NO environment of neighboring *L*-type Ca^2+^ channels.

Second, NO acts through two major types of signaling: rapid, reversible S-nitrosylation of protein thiols and the classical NO-sGC-cGMP pathway. The preferential choice of one pathway over the other is determined by redox status, availability of oxygen, and cofactors such as tetrahydrobiopterin (BH_4_) [24]. Third, the cellular context (species, developmental stage, experimental approach) is crucial for both channel expression and the machinery that orchestrates the upstream regulation. Together, these factors explain the conditional facilitation of, or decrease in, *I*_Ca,L_ by NO [25].

Our findings indicate that mechanical stimulation modulates L-type Ca^2+^ channel function by a fine-tuned interplay between S-nitrosylation and sGC-cGMP signaling. ODQ- and AA-treatment experiments lend further support to crosstalk processes in our system and indicate S-nitrosylation to be functionally relevant. Moreover, the differential effects of various NO donors suggest that the local activity of NO synthase (NOS)—particularly endothelial (eNOS) and/or neuronal NOS (nNOS)—is target-specific, probably depending on the nature of the protein substrates or the availability of reactive cysteine residues [26]. The phenomenon of transnitrostylation, in which intermediate proteins accept nitrosyl groups, complicates the issue even further [27]. Furthermore, S-nitrosylation itself may also suppress NOS activity, suggesting complex feedback regulation in the NO signaling system [28]. However, the specific spatial and molecular dynamics of these interactions are not fully elucidated.

NO has been demonstrated to be generated locally when the heart undergoes mechanical stretching, demonstrating the site specificity of NO release in ventricular cardiomyocytes [5]. This compartmentalization is essential for efficient ion channel regulation, particularly NOS3-mediated regulation. Moreover, NOS3^−/−^ mice are unable to produce stretch-induced NO responses, unlike NOS1^−/−^ and NOS2^−/−^ animals [29,30]. Approximately 20% of total NOS3 expression in the heart is derived from ventricular myocytes [31], highlighting its important role in mechanotransduction.

These findings underscore the importance of accurate spatial and temporal measurement of NO. The development of new techniques, such as biotin-switch assays, S-nitrosoproteomics, and fluorescent NO biosensors [32,33], has provided the opportunity to study S-nitrosylation and localization during mechanical manipulations. Moreover, cell-type specificity of this transduction pathway is indicated by varying NO production in cardiomyocytes and fibroblasts [34].

An additional complexity is related to the interaction between NO and *β*-adrenergic signaling. NO-releasing compounds have been reported to suppress isoprenaline-induced *I*_Ca,L_ in atrioventricular node (AVN) cells [32], suggesting coordinated regulation during mechanical and neurohumoral stress. This interaction may be responsible for adaptation of cardiac excitability to varying physiological demands. Such differences between studies could be due to variation in NO donor, redox status, phosphorylation, and/or NOS isoform contributions [35]. The cell response to NO is also dependent on developmental stage, cell type (atrial vs. ventricular), and metabolic condition [35,36].

Therefore, the relative significance of NO and the extent to which NO produces distinct outcomes vary in response to specific signaling parameters, so effects of NO must be interpreted within this context to reconcile discrepancies between reported data and their translational relevance.

### 3.3. Integration with Cellular Ca^2+^ Handling

The interrelationship between stretch-induced ionic currents and Ca^2+^ handling appears well tuned. 1,2-Bis(o-aminophenoxy) ethane-N,N,N’,N’-tetraacetic acid (BAPTA) does not block stretch-activated currents but alters mechanical effects on *I*_Ca,L_ [19]. It has also been found that BAPTA-AM was capable of inhibiting the stretch-induced decrease in *I*_Ca,L_ in guinea pig ventricular myocytes, implying the mediation of Ca^2+^-dependent inactivation in the mechanical regulation of *L*-type Ca^2+^ channels [37].

The stretch-induced regulation of *I*_Ca,L_ occurs most likely in combination with Ca^2+^-handling. Mechanical distortion may induce an increase in the intracellular Ca^2+^ level by a direct influx through MSCs [38] or by release of the Ca^2+^ from the sarcoplasmic reticulum [6]. The decrease of *I*_Ca,L_ upon stretch (Figure 2) may therefore be a protective feedback mechanism that restrains Ca^2+^ overload and ensures intracellular homeostasis under mechanical load.

### 3.4. Regulation Through Multiple Pathways

The intricate interaction between mechanical tension and Ca^2+^ channel activity consists of several, although partly redundant, signaling steps. The NO-sGC-cGMP system is not the only mediator of the stretch-induced changes, although it may play a significant role. Mechanical forces may also influence other ion transport pathways such as the Na^+^/Ca^2+^ exchanger (NCX), particularly if stretch-activated channels are also the site of enhanced Na^+^ influx and a rise in intracellular Na^+^ [39]. Furthermore, some K^+^ channels are also mechanosensitive and can potentially sense changes in intracellular Ca^2+^ or Na^+^ concentration [40].

Gadolinium (Gd^3+^), which has been commonly used as a non-specific blocker, provides valuable information about mechanosensitivity in a larger context. It has been demonstrated to block not only stretch-activated channels but also *L*-type Ca^2+^ channels, BK_Ca_ channels [41], and delayed rectifier potassium currents [42]. These wide-range inhibitory effects indicate that mechanosensitivity may be a more prevalent feature of ion channels than has been generally recognized.

The diversity of responses to SNAP in unstretched cells probably reflects the heterogeneity of either Ca^2+^ channel subtypes or of their regulatory environment. A similar variety of responses to SIN-1 has been described with either stimulation or inhibition of the Ca^2+^ current (*I*_Ca,L_) in guinea pig ventricular myocytes stimulated with isoprenaline [43] and in ferret ventricular myocytes [44]. This heterogeneity might thus allow fine-tuned contextual modulation of cardiac responses to mechanical input.

It should be noted that while this study concentrates on *L*-type Ca^2+^ channels, the cardiac action potential is maintained by the combined activity of a number of types of ion channels [45]. Voltage-sensitive sodium channels (Na_V_1.5) and numerous potassium channel isoforms, such as TREK-1, K_ATP_, and delayed rectifier channels, are also modulated by mechanical stretch and shape action potential morphology and conduction [46]. For instance, mechanical stimulation might increase late Na^+^ current or indirectly modulate NCX through intracellular Na^+^ buildup [47]. Mechanosensitive K^+^ channels, for their part, may abbreviate action potential duration, and thus, could work with lower *I*_Ca,L_ to prevent Ca^2+^ overload [48].

Taken together, the interaction and crosstalk of these mechanosensitive ion channels—particularly under disease-stretch or neurohumoral activation—present a major challenge in understanding the mechano-electrical feedback in the heart. Subsequent investigations should focus on decoding such interactions at the molecular and functional levels to fully appreciate their roles in health and disease.

### 3.5. Limitations and Future Directions

Although this research has expanded our knowledge about the mechanosensitive properties of *L*-type Ca^2+^ channels, several limitations need to be considered. While isolated rat ventricular cardiomyocytes are valuable for manipulating experimental conditions, they do not fully replicate the complexity of the whole working heart, where mechanical signaling is integrated within the multicellular network and influenced by neurohumoral factors. For enhanced translational relevance, future studies could integrate whole-heart models or human induced pluripotent stem cell-derived cardiomyocytes (hiPSC-CMs).

Our pharmacological data with ODQ, SNAP, AA, and NEM strongly suggest that both S-nitrosylation and NO-sGC-cGMP signaling are involved in stretch-induced modulation of *I*_Ca,L_ but the conclusions remain inferential, as no direct biochemical evidence for post-translational modification of Ca_V_1.2, such as S-nitrosylation, is present. The involvement of individual NOS isoforms is also unclear. Further studies utilizing direct detection techniques (e.g., biotin-switch assays) or isoform-specific knockout models are required to definitively determine the molecular basis for stretch-dependent regulation.

Furthermore, the time and location of NO signaling are still poorly defined, and there is a need to focus on compartmentalizing S-nitrosylation and cGMP pathways. Advanced real-time imaging techniques and biosensors will be indispensable in dissecting these interactions in subcellular compartments.

A more global perspective of cardiac mechanotransduction would also necessitate analyzing the crosstalk between the mechanosensitive Ca^2+^, Na^+^, and K^+^ channels. Mechanical modulation of late Na^+^ current and (or) NCX activity and (or) different K^+^ conductances could participate in synergy with *L*-type Ca^2+^ channel regulation to determine the final electrophysiological response to mechanical stress. These interactions should be viewed as integrated mechanisms rather than single components. Subsequent studies need to investigate these interactions together rather than individually.

Moreover, our patch-clamp protocol employed low EGTA (0.01 mmol/L) to maintain near physiological intracellular Ca^2+^ changes. Although this approach allows Ca^2+^-dependent inactivation and signaling to occur, it may confound the distinction between direct mechanical effects and Ca^2+^-mediated secondary responses. Complementary studies using higher concentrations of rapid Ca^2+^ buffers, such as BAPTA, could help isolate the contribution of direct mechanical gating.

Finally, it is intriguing to note that the targeting of NO-mediated mechanosensitive pathways may be a promising therapeutic strategy for cardiac diseases that requires further investigation. Such signaling circuits may be modulated by approaches that control them under pathologies associated with altered mechanical load and redox state, such as heart failure and hypertrophic cardiomyopathy.

In conclusion, this investigation provides novel insight into the NO-mediated mechano-electrical feedback in cardiomyocytes; however, future studies are required for a comprehensive understanding of the molecular basis, to confirm results in humans, and to determine therapeutic utility.

## 4. Materials and Methods

### 4.1. Animals and Cardiomyocyte Isolation

Male Wistar rats (8 weeks old, 180–200 g) were housed under standard conditions (12:12 h light:dark cycle) with ad libitum access to food. All experiments complied with the Guide for the Care and Use of Laboratory Animals (8th edition, 2011) and were approved by the institutional Ethics Committee.

Rats were anesthetized with ketamine (80 mg/kg) and xylazine (10 mg/kg) with heparin (1000 U/kg) added to prevent blood coagulation. Hearts were rapidly excised and mounted on a Langendorff apparatus for retrograde perfusion at 37 °C. Initial perfusion used Ca^2+^-free physiological salt solution (PSS) containing (in mmol/L): 118 NaCl, 4 KCl, 1 MgCl_2_, 1.6 NaH_2_PO_4_, 24 NaHCO_3_, 5 sodium pyruvate, 20 taurine, and 10 glucose (pH 7.4, carbogen-bubbled). After 5 min, hearts were perfused with enzyme medium (Ca^2+^-free PSS supplemented with 10 μmol/L CaCl_2_, 0.2 mg/mL type II collagenase, and 1 mg/mL BSA) for 18–20 min. Ventricles were then excised, cut into strips, and mechanically dissociated in modified Kraftbrühe (KB) medium.

### 4.2. Patch-Clamp Recordings

Whole-cell patch-clamp recordings were performed at 37 °C using borosilicate glass electrodes (1.8–2.2 MΩ). The external solution contained (mmol/L): 150 NaCl, 5.4 KCl, 1.8 CaCl_2_, 1.2 MgCl_2_, 20 glucose, and 5 HEPES (pH 7.4). The internal solution contained (mmol/L) 140 KCl, 5 Na_2_ATP, 5 MgCl_2_, 0.01 EGTA, and 10 HEPES/KOH (pH 7.3). Currents were recorded using an Axopatch 200B amplifier (Molecular Devices, Sunnyvale, CA, USA) and pClamp 10 software, filtered at 2 kHz and sampled at 5 kHz. In K^+^_in_/K^+^_out_ solutions, the current through L-type Ca^2+^ channels (*I*_Ca,L_) was estimated as the difference between the negative peak Ca^2+^ current and the late current (*I*_L_) in control conditions [49,50]. Whole-cell patch-clamp recordings were performed using an internal solution containing (in mmol/L): 130 KCl, 1 MgCl_2_, 5 Na_2_ATP, 0.01 EGTA, and 10 HEPES; pH adjusted to 7.2 with KOH. A low EGTA concentration was used to permit physiological Ca^2+^ dynamics and avoid artificial suppression of Ca^2+^-sensitive mechanisms.

In addition, cells with Rs values exceeding 4 MΩ at any point during the experiment were excluded from the final analysis to minimize potential voltage-clamp errors and to ensure the accuracy of current–voltage relationships, particularly under mechanical stretch conditions. Data were digitized at 10 kHz and filtered at 2 kHz.

### 4.3. Mechanical Stretch Protocol

Local axial stretch was applied using a fire-polished glass stylus (diameter 14 ± 0.8 µm) controlled by a motorized micromanipulator (MP 285, Sutter, accuracy 0.2 µm). The stylus and patch pipette were positioned 40 µm apart at 45° angles to the glass bottom. Sarcomere length was measured using an Olympus XM10 camera and CellSens (Olympus Corporation, Tokyo, Japan), version 1.18, (https://www.olympus-lifescience.com/en/software/cellsens/; accessed on 1 April 2025), before stretch (1.83 ± 0.01 µm) and during stretches of 4–10 µm.

### 4.4. Pharmacological Interventions

S-nitroso-N-acetylpenicillamine (SNAP) (200 µmol/L) was used as an NO donor. 1H-[1,2,4]Oxadiazolo[4,3-a]quinoxalin-1-one (ODQ) (5 µmol/L) was employed to inhibit sGC. Ascorbic acid (AA) (10 μmol/L) was used to inhibit S-nitrosylation. N-ethylmaleimide (NEM), (200 µmol/L) was applied as a thiol-alkylating agent at 22 °C due to temperature-dependent toxicity. All compounds were prepared fresh before experiments.

### 4.5. RNA Sequencing

RNA was isolated from cardiomyocytes using TRIzol followed by chloroform extraction and RNeasy mini kit purification. RNA quality was assessed using NanoDrop (Thermo Fisher Scientific, Waltham, MA, USA) and the Qi-RNA kit (Qiagen, Hilden, Germany). Libraries were prepared using the NEB Ultra II RNA kit (New England Biolabs, Ipswich, MA, USA) with NEBNext Poly(A) magnetic isolation and unique dual-indexing. Sequencing was performed on an Illumina NovaSeq 6000 (2 × 150 bp paired-end), (Illumina, San Diego, CA, USA). Raw reads were quality-checked using FastQC v0.11.5, trimmed with Trimmomatic v0.36, and aligned to the rat reference genome (mRatBN7.2) using HISAT2 (version 2.2.1), https://daehwankimlab.github.io/hisat2/ (accessed on 1 March 2025) [51,52,53,54]. Expression levels were calculated as transcripts per million (TPM).

### 4.6. Data Analysis

Data were analyzed using pClamp 10.2 software. For statistical comparisons, *p*-values are reported as exact values where possible. One-way repeated measures ANOVA with the Holm–Sidak post hoc test was used, and comparisons are described using group names (e.g., control, stretch, drug treatment) for clarity rather than arbitrary lettering. Normality was verified using the Shapiro–Wilk test. Data are presented as mean ± SEM, with n representing the number of cells (249) and m representing the number of rats (114).

## 5. Conclusions

We show that *I*_Ca,L_ is decreased by mechanical stretch in cardiomyocytes and that this effect is mediated by NO production through both S-nitrosylation and the NO-sGC-cGMP pathway. The opposing effects of NO donors and inhibitors indicate a more complex regulatory network that involves overlapping, context-sensitive mechanisms. Such findings provide further insights into cardiac mechano-electrical coupling and have potentially wide-ranging implications in settings where mechanical stress is a major driver of pathology, such as in infarction, heart failure, and hypertrophy.

From a translational perspective, selective intervention in NO signaling might represent new therapeutic options in diseases involving mechanically mediated stimuli. Pharmacological manipulation of both S-nitrosylation and cGMP-dependent signaling might be expected to enhance Ca^2+^ handling and performance of the heart during mechanical stress.

Future studies will also need to determine the molecular interactions between NO signaling components and Ca_V_1.2 and their spatial and temporal properties in health and disease. In human cardiomyocytes, including those from induced pluripotent stem cells, studies will be required to validate these mechanisms and evaluate their clinical applicability.

Although the idea of targeting NO-mediated mechanosensitivity is at an exploratory stage, this research provides a mechanistic framework for further exploration. Additional disease models and in vivo studies are required to evaluate the therapeutic potential of modulating the stretch-sensitive Ca^2+^ channel. However, our results provide a platform for further translational research on the mechanical regulation of cardiac function.

## Data Availability

The data that support the findings of this study are available in Gene Expression Omnibus (GEO) at https://www.ncbi.nlm.nih.gov/geo/query/acc.cgi?acc=GSE285899 (accessed on 1 March 2025). All further data gathered in this study (including manual patch-clamp measurements and their analysis procedures) are available from the corresponding author upon request.

## Abbreviations

AA	Ascorbic Acid
ATP	Adenosine Triphosphate
BAPTA	1,2-Bis(o-aminophenoxy)ethane-N,N,N′,N′-Tetraacetic Acid
BSA	Bovine Serum Albumin
CMs	Cardiomyocytes
cGMP	Cyclic Guanosine Monophosphate
EGTA	Ethylene Glycol Tetraacetic Acid
GEO	Gene Expression Omnibus
hiPSC-CMs	Human-Induced Pluripotent Stem Cell–Derived Cardiomyocytes
*I*_Ca,L_	L-type Calcium Current
iPSCs	Induced Pluripotent Stem Cells
IV curve	Current–Voltage Curve
KB medium	Kraftbrühe Medium
MGCs	Mechanically Gated Channels
mRNA	Messenger Ribonucleic Acid
MSCs	Mechano-Sensitive Channels
NEB	New England Biolabs
NEM	N-Ethylmaleimide
NO	Nitric Oxide
NOS	Nitric Oxide Synthase
NOS1/2/3	Nitric Oxide Synthase Isoforms (neuronal, inducible, endothelial)
ODQ	1H-[1,2,4]Oxadiazolo[4,3-a]quinoxalin-1-one
PSS	Physiological Salt Solution
RNA	Ribonucleic Acid
ROS	Reactive Oxygen Species
RT-qPCR	Real-Time Quantitative Polymerase Chain Reaction (if mentioned, inferred from context)
sGC	Soluble Guanylyl Cyclase
SEM	Standard Error of the Mean
SIN-1	3-Morpholinosydnonimine
SNAP	S-nitroso-N-acetylpenicillamine
TPM	Transcripts Per Million
TRIzol	Commercial Reagent for RNA Isolation
